# Selenium Modulates the Level of Auxin to Alleviate the Toxicity of Cadmium in Tobacco

**DOI:** 10.3390/ijms20153772

**Published:** 2019-08-01

**Authors:** Yong Luo, Yuewei Wei, Shuguang Sun, Jian Wang, Weifeng Wang, Dan Han, Huifang Shao, Hongfang Jia, Yunpeng Fu

**Affiliations:** 1 National Tobacco Cultivation & Physiology & Biochemistry Research Centre, College of Tobacco Science, Henan Agricultural University, Zhengzhou 450002, China; 2 China Tobacco Hubei Industrial Co., Ltd., Wuhan 430040, China; 3 Guangxi Zhuang Autonomous Region Provincial Branch of China National Tobacco Corporation, Nanning 530000, China

**Keywords:** selenium, cadmium stress, auxin, root architecture, phosphate transporter, *Nicotiana tabacum*

## Abstract

Cadmium (Cd) is an environmental pollutant that potentially threatens human health worldwide. Developing approaches for efficiently treating environmental Cd is a priority. Selenium (Se) plays important role in the protection of plants against various abiotic stresses, including heavy metals. Previous research has shown that Se can alleviate Cd toxicity, but the molecular mechanism is still not clear. In this study, we explore the function of auxin and phosphate (P) in tobacco (*Nicotiana tabacum*), with particular focus on their interaction with Se and Cd. Under Cd stress conditions, low Se (10 μM) significantly increased the biomass and antioxidant capacity of tobacco plants and reduced uptake of Cd. We also measured the auxin concentration and expression of auxin-relative genes in tobacco and found that plants treated with low Se (10 μM) had higher auxin concentrations at different Cd supply levels (0 μM, 20 μM, 50 μM) compared with no Se treatment, probably due to increased expression of auxin synthesis genes and auxin efflux carriers. Overexpression of a high affinity phosphate transporter NtPT2 enhanced the tolerance of tobacco to Cd stress, possibly by increasing the total P and Se content and decreasing Cd accumulation compared to that in the wild type (WT). Our results show that there is an interactive mechanism among P, Se, Cd, and auxin that affects plant growth and may provide a new approach for relieving Cd toxicity in plants.

## 1. Introduction

Cadmium (Cd) is a highly toxic heavy metal, which is widely distributed in the environment [1]. In recent years, industrial and agricultural production have discharged Cd to varying degrees, and it has become one of the most widely distributed agricultural pollutants [2]. Cd is not needed for plant growth and development but is more likely to accumulate in plants than other heavy metals [3]. Evidence suggests that a high concentration of Cd in soil affects the growth and development of plants through physiological and biochemical processes, including inhibition of plant enzyme and membrane activity [4], decreased cell division [5], reduced growth rate [6], damaged photosynthesis [7], inhibition of stomatal opening [8], and promotion of lipid peroxidation [9]. Cd bioaccumulates and in humans can cause diseases such as osteoporosis, anemia, hypertension, and kidney damage. Soil pollution by Cd has become a serious threat to the safety of agricultural produce. To address this issue, it is important to develop a comprehensive understanding of the mechanism of Cd uptake in plants. Selenium (Se) is not essential for plant nutrition, but it can play a beneficial role in plant health. A suitable dose of Se can enhance antioxidant capacity, delay aging, increase photosynthesis, boost auxin content, and promote plant growth [10,11]. By contrast, a high dose of Se can damage plants through reactive oxygen species (ROS) accumulation and inhibit plant growth [12]. Plants are able to utilize soil Se in its inorganic Se (IV) and Se (VI) forms (selenite and selenate, respectively). Studies have shown that plants absorb selenate through sulfate transporters and selenite through phosphate channels [13,14]. A previous study has suggested that Se (IV) uptake is mediated by Pi (inorganic phosphate) transporters [15]. Recent research suggests that auxin is involved in the interaction between Pi and Se in tobacco, which provides convincing evidence for understanding the molecular mechanism of how Se regulates plant growth [16].

Se inhibits Cd uptake, which can relieve the toxic effects of Cd in plants. Cary et al. (1981) first reported that Se fertilizer reduced the absorption of Cd in wheat and lettuce [17]. Increasing numbers of studies have shown that there is antagonism between Cd and Se in plants [18,19,20]. Cd stress has also been shown to inhibit root growth and leaf photosynthesis in winter wheat, although adding some doses of Se can alleviate the Cd toxicity by enhancing root growth [21]. Despite studies investigating the antagonistic relationship between Se and Cd, this relationship—especially the molecular mechanism of Se remission of toxic Cd effects—is still not clear.

Tobacco is an important economic and model crop. In this study, we aimed to clarify the molecular mechanism of Se remission of toxic Cd effects in tobacco. We chose *DR5::GUS* and *NtPT2* overexpressed transgenic tobacco as the test material. We studied the influence of Cd on growth; the antioxidant system; the auxin distribution; and the Se, Cd uptake of tobacco under various Se and Cd treatments. We aimed to (1) clarify the function of auxin in tobacco growth, under different doses of Se and Cd; (2) determine the mechanism by which Se enhances tolerance to Cd stress; and (3) reveal the function of phosphate transporter (*NtPT2*) in the interaction of Se and Cd in plants.

## 2. Results

### 2.1. Effects of Se on Tobacco Phenotype and Biomass under Cd Stress

**Figure 1:**
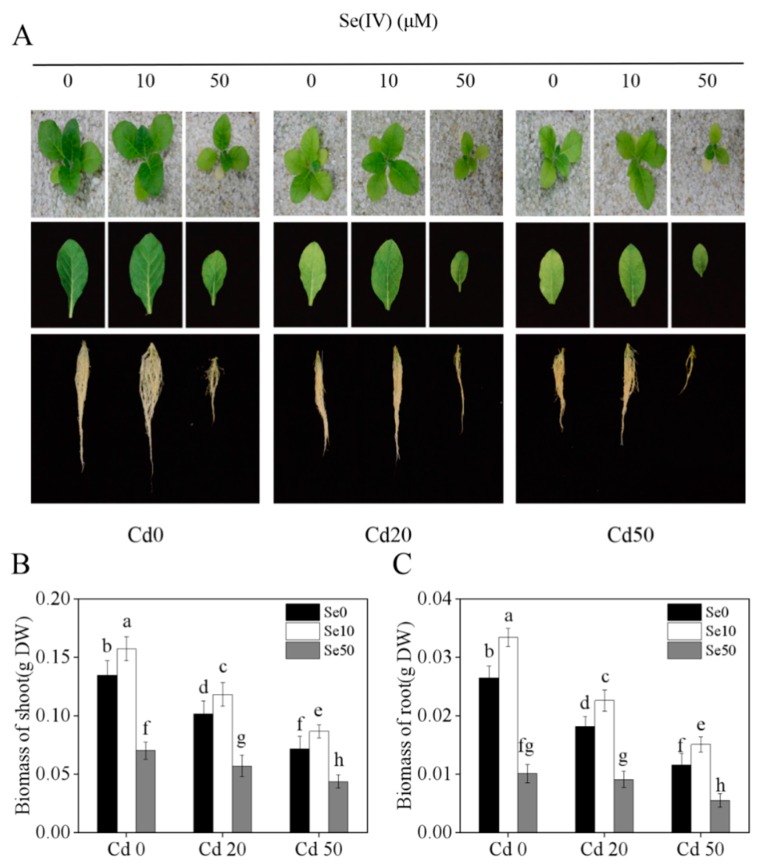
Characterization of tobacco under different Se and Cd concentration supply conditions for 21 days. (**A**) The phenotype of shoot, third young leaf and root under Se (Ⅳ) and Cd (Ⅱ) treatments in tobacco; (**B**) The biomass of shoot under different Se (Ⅳ) and Cd (Ⅱ) concentration supply conditions; (**C**) The biomass of root under different Se (Ⅳ) and Cd (Ⅱ) concentration supply conditions. 14-days-old seedlings (wild-type, Yunyan 87) were grown in pots with sand under different Se (0, 10, 50 μM) and Cd (0, 20, 50 μM) concentrations for 21 days. **Se0**: no Se; **Se10**: Se, 10 μM; **Se50**: Se, 50 μM; **Cd0:** no Cd; **Cd20**: Cd, 20 μM; **Cd50**: 50 μM. Shown are mean ± standard deviation (SD) from five biological replicates. DW, dry weight. Different letters indicate significant differences (*p* < 0.05).

To determine whether Se affects the growth of tobacco under different Cd treatments, we checked the characterization of tobacco plants. We found that Cd treatment had a substantial toxic effect on tobacco seedlings, with greater yellow leaf area and more poorly developed root architecture in Cd-treated seedlings than those in the Cd0 treatment (Figure 1A and Figure S1). Interestingly, we also found that low Se (Se10) could promote the growth of tobacco under Cd stress (Figure 1A); under Cd20 and Cd50 stress conditions, the biomass of shoots with Se10 treatments increased by 16.3% and 20.8%, respectively, compared to those with no Se added (Figure 1B), and the biomass of roots with Se10 treatments increased by 24.2% and 30.2%, respectively (Figure 1C). By contrast, high Se (Se50) levels suppressed tobacco growth under different Cd (Cd0, Cd20 and Cd50) treatments (Figure 1). These results show that Se has a dual effect on Cd stress, and the appropriate concentration of Se can alleviate Cd toxicity in tobacco.

### 2.2. Effects of Se and Cd Interactions on Tobacco Antioxidant Capacity

**Figure 2:**
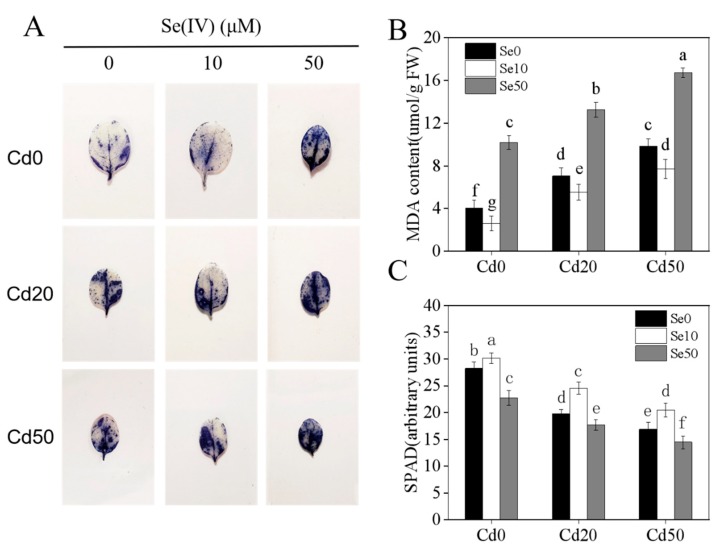
Effects of Se and Cd treatments on antioxidant capacity of tobacco. (**A**) nitroblue tetrazolium (NBT) staining of tobacco seedlings under different Se (Ⅳ) and Cd (Ⅱ) concentration supply conditions; (**B**) the content of MDA of tobacco seedlings under different Se (Ⅳ) and Cd (Ⅱ) concentration supply conditions; (**C**) the SPAD of the third young leaf of tobacco seedlings under different Se (Ⅳ) and Cd (Ⅱ) concentration supply conditions; 14-days-old seedlings (wild-type, Yunyan 87) were grown in pot with sand under different Se (0, 10, 50 μM) and Cd (0, 20, 50 μM) concentrations for 21 days. **Se0**: no Se; **Se10**: Se, 10 μM; **Se50**: Se, 50 μM; **Cd0**: no Cd; **Cd20**: Cd, 20 μM; **Cd50**: Cd, 50 μM. Shown are mean ± SD from five biological replicates. Different letters indicate significant differences (*p* < 0.05).

Previous studies showed that Se can enhance the enzymatic and non-enzymatic anti-oxidation systems and improve plant resistance to abiotic stresses in plants [4,5,6,7]. To determine the function of Se under Cd stress conditions, we measured the antioxidant capacity and chlorophyll content of tobacco. Firstly, we determined the accumulation of H_2_O_2_ by nitroblue tetrazolium (NBT) staining. Low Se (Se10) obviously reduced the accumulation of H_2_O_2_ (Figure 2A), which was consistent with the results of Malondialdehyde (MDA) content under Cd stress conditions (Figure 2B). We also checked the chlorophyll content, and found that Se had notable effects on chlorophyll content in the leaves (Figure 2C). At no Se (Se0) and high Se (Se50) levels, increased Cd levels significantly reduced tobacco chlorophyll content. Notably, the chlorophyll content of the low Se (Se10) treatment showed a remarkable increase under different Cd levels, which implies that low Se can promote tobacco growth under Cd stress by improving the anti-oxidation activity of tobacco plants.

### 2.3. Accumulation of Se and Cd in Tobacco

**Figure 3:**
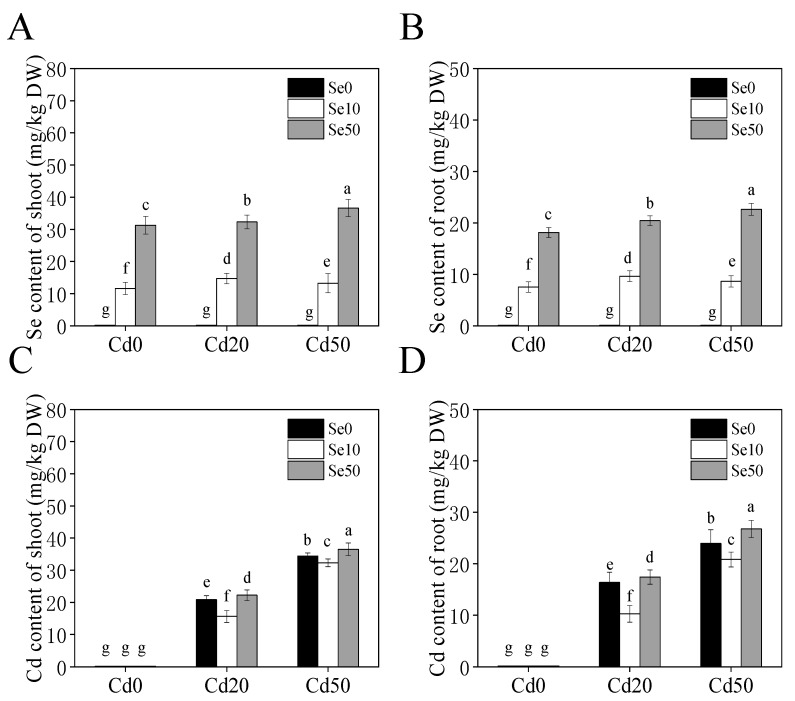
Content of Se and Cd in tobacco under different Se and Cd concentration supply conditions for 21 days. (**A**,**B**) Se content of the shoots and roots under different Se (Ⅳ) and Cd (Ⅱ) concentration supply conditions; (**C**,**D**) Cd content of the shoots and roots under different Se (Ⅳ) and Cd (Ⅱ) concentration supply conditions. 14-days-old seedlings (wild-type, Yunyan 87) were grown in pot with sand under different Se (0, 10, 50 μM) and Cd (0, 20, 50 μM) concentration for 21 days. **Se0**: no Se; **Se10**: Se, 10 μM; **Se50**: Se, 50 μM; **Cd0**: no Cd; **Cd20**: Cd, 20 μM; **Cd50**: Cd, 50 μM. Shown are mean ± SD from five biological replicates. Different letters indicate significant differences (*p* < 0.05).

To investigate the accumulation of Cd in tobacco under low Se (Se10) and high Se (Se50) conditions, we monitored the Se and Cd content in the roots and shoots of tobacco seedlings (Figure 3). Under high Se and low Se conditions, the Se content of the shoots and roots increased in Cd20 and Cd50 treatments compared with the Cd0 treatment (Figure 3A,B). Under Cd stress (Cd20, Cd50) conditions, the low Se treatment significantly reduced the Cd content of tobacco shoots and roots, especially under the Cd20 treatment, where the root Cd content was 37.3% lower than that observed with the Se0 treatment (Figure 3C,D). We also found that high Se did not reduce the Cd content of tobacco plants, suggesting that variation in Se content can affect the uptake of Cd in tobacco.

### 2.4. Effects of Se on Auxin and Expression of Auxin-Related Genes in Tobacco under Cd Stress

**Figure 4:**
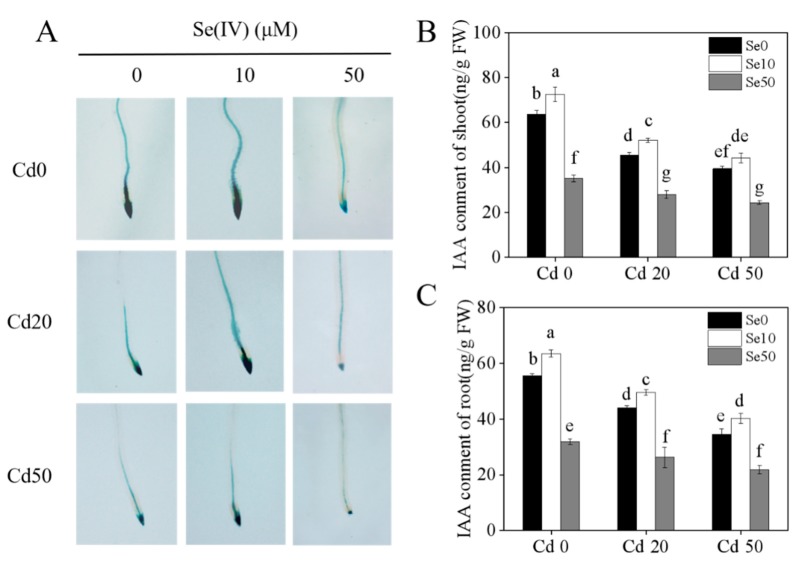
Histochemical localization of *DR5::GUS* and indole-3-acetic acid (IAA) contents of tobacco under different Se and Cd concentration supply conditions. (**A**) Histochemical localization of *DR5::GUS* in root tips of tobacco under different Se (Ⅳ) and Cd (Ⅱ) concentration supply conditions; (**B**,**C**) IAA content of the shoots and roots under different Se (Ⅳ) and Cd (Ⅱ) concentration supply conditions. 14-days-old seedlings (*DR5::GUS* transgenic tobacco) were grown in pot with sand under different Se (0, 10, 50 μM) and Cd (0, 20, 50 μM) concentrations for 21 days. **Se0**: no Se; **Se10**: Se, 10 μM; **Se50**: Se, 50 μM; **Cd0:** no Cd; **Cd20:** Cd, 20 μM; **Cd50**: Cd, 50 μM. Shown are mean ± SD from five biological replicates. Different letters indicate significant differences (*p* < 0.05).

To investigate whether auxin is involved in the growth of tobacco roots under Se and Cd treatment, we used *DR5::GUS* transgenic tobacco, which could reflect the distribution of auxin in the plant [22,23,24]. We detected *GUS* expression in the root tip under Se and Cd treatments (Figure 4A). Under Cd stress (Cd20, Cd50) conditions, the *GUS* expression in the root tip of plants was much lower than observed in the Cd0 treatment. Under low Se (Se10), the expression of *GUS* in the root tip was much greater than that in Se0 tobacco. Low Se could increase the expression of *GUS* under Cd stress. We also checked the auxin content of shoots and roots, which was consistent with the *GUS* expression results (Figure 4B,C).

**Figure 5:**
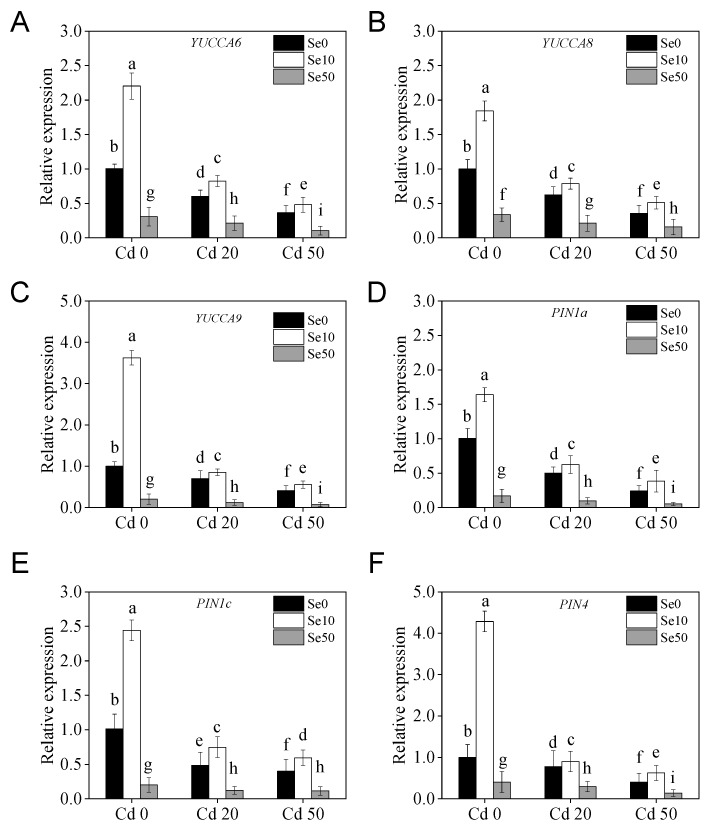
Expression of auxin-relative gene in tobacco under different Se and Cd concentration supply conditions. (**A**–C) Expression of three members (*YUCCA6, 8, 9*) of the tobacco *YUCCAs* family genes in shoots under different Se and Cd concentration supply conditions.; (**D**–**F**) Expression of three members (*PIN1a, 1c, 4*) of the tobacco *PINs* family genes in roots under different Se and Cd concentration supply conditions. 14-days-old seedlings (*DR5::GUS* transgenic tobacco) were grown in pot with sand under different Se (0, 10, 50 μM) and Cd (0, 20, 50 μM) concentrations for 21 days. The tobacco housekeeping gene *L25* was used as an internal control. The relative expression levels are shown compared with the expression under Cd0 and Se0 (Cd0 + Se0) conditions as 1 expression. **Se0**: no Se; **Se10**: Se, 10 μM; **Se50**: Se, 50 μM; **Cd0**: no Cd; **Cd20**: Cd, 20 μM; **Cd50**: Cd, 50 μM. Shown are mean ± SD from five biological replicates. Different letters indicate significant differences (*p* < 0.05).

Local auxin levels are determined by biosynthesis and intercellular transport in plant roots [25]. To detect whether Se and Cd treatments affect the auxin-signal pathway, we analyzed the expression of *YUCCAs* and *PINs* family genes, which are involved in auxin biosynthesis and transport in tobacco under different Se and Cd treatments. Under Cd stress conditions, the expression of *NtYUCCA 6*, *8*, and *9*, and *NtPIN 1a*, *1c*, and *4* was substantially higher under low Se treatments, which was consistent with the auxin content results (Figure 5A–F). All these results suggest that auxin may play a key role in growth under Se and Cd treatment conditions.

### 2.5. Overexpression of NtPT2 Could Enhance the Tolerance of Cd Stress under Low Se Conditions

**Figure 6:**
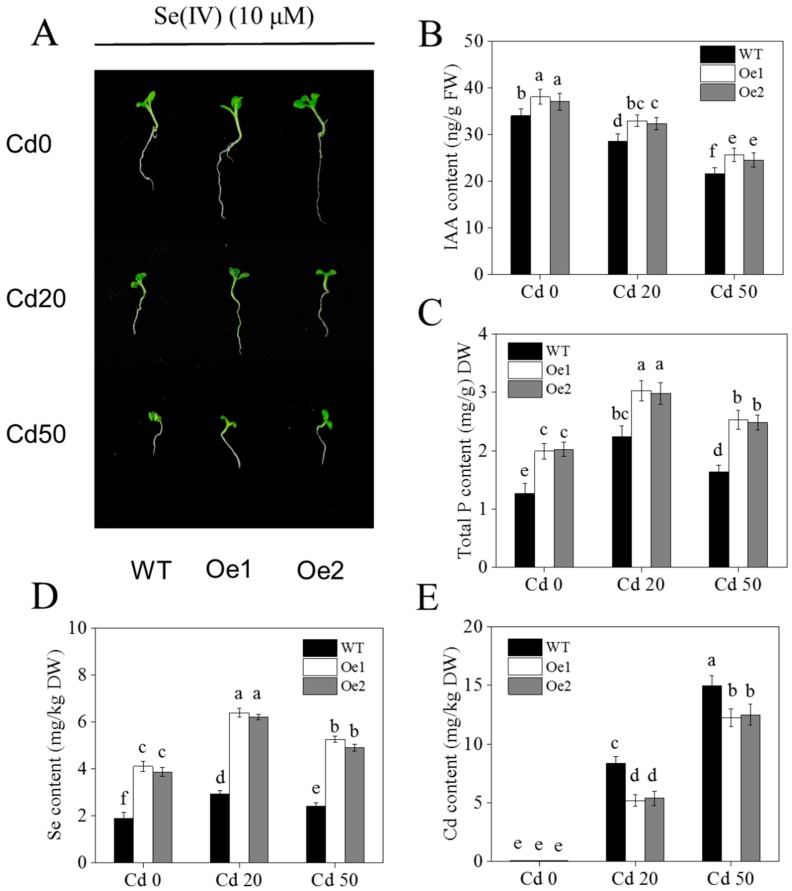
Effects of Cd on *NtPT2-Oe* transgenic tobacco under different Se and Cd concentration supply conditions. (**A**,**B**) The phenotype and IAA content of *NtPT2-Oe* transgenic tobacco seedlings in Se (10 μM) and different Cd (0, 20, 50 μM) concentrations supply conditions; (**C**–**E**) Total P, Se and Cd content of the whole transgenic plant. Tobacco seeds were grown in 1/2MS culture under Se (10 μM) and different Cd (0, 20, 50 μM) concentrations for 14 days. **Se0**: no Se; **Se10**: Se, 10 μM; **Se50**: Se, 50 μM; **Cd0**: no Cd; **Cd20**: Cd, 20 μM; **Cd50**: Cd, 50 μM. Shown are mean ± SD from five biological replicates. Different letters indicate significant differences (*p* < 0.05).

Phosphate transporters are not only involved in Pi uptake, but also in selenite uptake [14,26]. Recently, we reported that the expression of a high-affinity phosphate transporter (*NtPT2*) involved in Se uptake in tobacco is induced in the roots and shoots under low Pi conditions [16]. In this study, we used *NtPT2* overexpression (*NtPT2-Oe*, two independent transgenic lines: Oe1 and Oe2) in transgenic tobacco to clarify the molecular mechanism by which Se can alleviate Cd toxicity. We found that *NtPT2* overexpression in plants enhanced their tolerance to Cd stress, with plants exhibiting better roots and shoots than the WT under Cd stress conditions. This was consistent with the auxin content differences between the *NtPT2-Oe* plant and the WT (Figure 6A,B and Figure S2). We also checked the P, Se, and Cd content in both *NtPT2-Oe* and WT plants; the total P and Se content of *NtPT2-Oe* plants was higher than that of the WT plants under Cd stress conditions (Figure 6C,D), suggesting that *NtPT2* is involved in P and Se uptake in tobacco. We also found that Cd content was significantly reduced in *NtPT2-Oe* plants under Cd stress conditions (Figure 6E), confirming that overexpression of *NtPT2* could enhance the tolerance of tobacco plants to Cd stress.

## 3. Discussion

### 3.1. Se Affects the Growth of Tobacco Roots by Changing Auxin Concentration under Cd Stress

Cd has high levels of biological toxicity and can inhibit the growth and development of plants [27]. Biomass is an important indicator of plant growth and development. In this study, we showed that under Cd stress conditions, the biomass of tobacco, especially in the roots, was significantly lower than plants not exposed to Cd stresses. This indicates that the roots and shoots were damaged by Cd stress, which is consistent with the results of Li et al. [28]. We also found that low Se levels stimulated growth in tobacco and could effectively alleviate the toxic effects of Cd stress (Figure 1), which is consistent with the results of previous studies [21,29]. Previous studies have shown that low Se enhances the antioxidation of enzymatic and non-enzymatic systems, changes root growth, and promotes absorption of nutrients [30,31], thus, improving the ability of plants to resist abiotic stresses. Low Se increased anti-oxidation activity (Figure 2), reduced the content of Cd (Figure 3) and changed root development by increasing auxin concentration in the roots (Figure 4), which may have increased growth. Further analyses on auxin-related genes showed that the expression levels of *YUCCAs* and *NtPINs* family genes were markedly higher under low Se conditions (Figure 5), indicating that low Se affects the growth of roots by changing auxin concentration under Cd stress.

Se often exerts a dual effect on plant growth. High levels of Se cause toxicity in plants, such as accumulation of ROS and inhibition of plant development [12,30,31]. A recent study showed that high Se levels inhibit root growth [16]. In this study, we also found high Se significantly decreased the biomass and auxin content of roots. Notably, under high Se conditions, the Cd treatments significantly decreased the biomass and auxin content in the shoots and roots. To investigate whether auxin is a major regulator of plant growth and development under Cd stress conditions, we also checked the characterization of root in *DR5::GUS* transgenic tobacco under different Se and Cd concentration supply conditions by adding IAA (100 nM). The results showed that exogenous IAA could increase the root biomass and the length of primary root, which implies that IAA plays a key roles in regulating the root architecture under Cd stress conditions (Figure S3).

Altogether, our data suggest that, under Cd stress conditions, Se might affect the growth and development of plants by changing auxin concentration. Not only the auxin pathway, but also other endogenous hormones (e.g., cytokinin, ethylene and gibberellin) may have an effect on plant root development. Recent studies showed that Se increases primary root length through alteration of the auxin and ethylene balance in rice, growth inhibition in Se-treated *Arabidopsis* is associated with an incomplete mobilization of starch, and high concentrations of selenite-induced enhancement of ethylene biosynthesis may result in plant cell death [32,33,34]. Therefore, the function of cytokinin, ethylene, gibberellin and other hormones in the development of tobacco roots under the treatment of Se and Cd need further study.

### 3.2. *NtPT2* Might be a Potential Candidate Gene for Breeding Cd-Tolerant Plants

A large number of genes encoding Pi transporters have been identified in different plant species. Pi transporters are generally classified into the *Pht1*, *Pht2*, *Pht3*, and PT gene families [35,36]. Most of the high-affinity Pi transporter (*Pht1*) family genes had induced expression under low Pi stress conditions, suggesting that *Pht1* plays a crucial role in both Pi uptake and translocation under Pi deficiency [37,38]. Recent studies have suggested that Se and Pi share similar uptake mechanisms and that Pi transporters are involved in Se uptake in plants, which might occur via a Se-H^+^ symport process in the plant cell membrane [14,16,26].

Although a large number of *Pht1* family genes have been studied in rice and *Arabidopsis*, the function of key Pi transporters in plants need further research. For instance, recently, two key phosphate transporter genes *OsPT2* and *OsPT8* were found to play a key role in As and Se uptake and translation in rice, implying that some phosphate transporters are involved in the uptake of heavy metals or trace elements [39]. At present, studies on the *Pht1* family gene in tobacco are few. In our previous work, we showed that *NtPT2* is the most closely related to *OsPT2,* and that *NtPT2* has similar expression patterns to *OsPT2* (low Pi-induced expression in roots) [16]. In this study, we used *NtPT2* overexpressing transgenic tobacco to further clarify whether Se could alleviate the toxicity of Cd. Our results showed that when 10 μM Se was supplied, the *NtPT2* overexpression significantly increased biomass, total P, axuin, and Se content. In contrast, the Cd content in transgenic tobacco obviously reduced under Cd treatments compared with WT (Figure 6), implying that a suitable level of Se modulates the level of auxin, enhancing the tolerance of tobacco to Cd stress.

With the improvement of health awareness, how to reduce the Cd content in crop production and food chain has become a research hotspot in recent years. It has a potential impact on reducing the accumulation of Cd in tissues such as liver, kidney, lungs and bones, and avoiding the induction of various diseases such as lung cancer, hypertension and cardiomyopathy [40,41]. Our results suggest that *NtPT2* might be a potential candidate gene for breeding Cd-tolerant plants, which also need further research.

**Figure 7:**
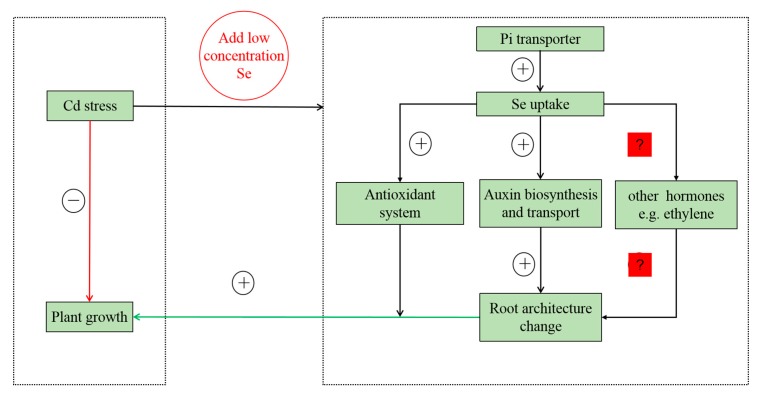
The model for the interaction mechanism between Se, Cd stress, *p* and auxin response in tobacco. The model is based on the results presented here. + indicates positive regulations and – indicates negative regulations. ? indicates that requires further research.

Based on this study, we propose a possible model for revealing the mechanism controlling the Se, Cd stress, P, and auxin response in tobacco (Figure 7). Under Cd stress conditions, (1) plant growth is inhibited. (2) Under low Se and Cd stress conditions, the Pi transporter is involved in the uptake of Se (IV) and P in the root, and low Se (accumulation of a small amount Se in plant) changes the auxin-related gene expression, which increases auxin content to promote plant growth. (3) Low Se not only increases the biomass of the root but also enhances the antioxidant capacity. We conclude that low Se alleviates Cd toxicity in tobacco. However, the function of other hormones in the development of tobacco roots under the treatment of Se and Cd needs further study.

## 4. Materials and Methods

### 4.1. Plant Material and Experimental Conditions

The tested materials were the wild-type (*Nicotiana tabacum* cv,Yunyan 87), *DR5::GUS* and *NtPT2-Oe* transgenic seeds (T2 generation) (Figure S4). The generation of transgenic tobacco material and the construction of *pDR5::GUS* had been detailed in the previous studies [16,26,42].

Tobacco seeds were sterilized in solution of 75% (*v/v*) ethanol for 30 s and 10% (*v/v*) sodium hypochlorite for 7 min, then followed by washing 6 times with sterile distilled water. The seeds were then transferred to seedling tray (3 days) kept in the culture room at 28 °C in dark for proper germination. Germinated seedlings were placed in greenhouse for 10 days. The culture during the first five days used one-fourth-strength Hoagland’s nutrient solution, and half-strength nutrient solution was used in the second five days. The tobacco seedlings were transferred in pot with sand and half-strength nutrient solution was used for 4 days in seedling recovering stage. Then they were exposed to Se (Na_2_SeO_3_) and Cd (CdCl_2_·2.5H_2_O) for 21 days. The experiment of three Se levels, i.e., 0, 10 and 50 μM, and three Cd levels, i.e., 0, 20 and 50 μM were designed. There was a total of nine treatments: Cd0+Se0, Cd0+Se10, Cd0+Se50, Cd20+Se0, Cd20+Se10, Cd20+Se50, Cd50+Se0, Cd50+Se10, and Cd0+Se50. Se and Cd were added into the nutrient solution in the forms of Na_2_SeO_3_ and CdCl_2_·2.5H_2_O, respectively.

The tobacco seedings were harvested after 21 days of treatment. Shoots and roots were washed with deionized water for further analysis. (1) We observed and recorded the phenotype of tobacco plants. (2) Some seedings were used to measure the IAA, chlorophyll and MDA content. (3) Some of the leaves and roots were used for NBT and *DR5::GUS* staining. (4) The other tobacco seedings were oven-dried and used to measure the contents of Se and Cd. (5) Some seedings were used for detecting the gene expression and enzyme activity.

For IAA (Indole-3-acetic acid, dissolved in 1 M NaOH) treatments, 100 nM IAA was added to the nutrient solution under different Se and Cd treatments, respectively. The nine treatment plants, namely Cd0+Se0+IAA, Cd0+Se10+IAA, Cd0+Se50+IAA, Cd20+Se0+IAA, Cd20+Se10+IAA, Cd20+Se50+IAA, Cd50+Se0+IAA, Cd50+Se10+IAA and Cd50+Se50+IAA. The tobacco seedings were grown in a growth chamber for 7 days. The plants were then harvested for next stage of analysis. (1) To observe the phenphtype of tobacco plants. (2) To record the histochemical localization of GUS.

### 4.2. GUS Staining and Nitroblue Tetrazolium (NBT) Staining of Plant Tissues

Plants were stained for GUS activity for 24 h at 37 °C, and then seedlings were immersed in 95% ethanol to eliminate chlorophyll pigmentation. Plants were stained in NBT solution for 5 h at 30 °C, and 95% ethanol was used until decolorization was complete. The stereo microscope (Olympus SZX16, Olympus, Tokyo, Japan) equipped with a colorcharge coupled device (CCD) camera were used to photograph stained plant tissues.

### 4.3. Chlorophyll and MDA Measurement

The relative amount of chlorophyll in plants was determined by a SPAD-502 chlorophyll meter (Konica, Tokyo, Japan) [43]. Five sites of one leaf were measured, and the results were averaged. The content of MDA was determined by the thio-barbituric acid method (TBA) which was calculated by using the difference in absorbance of the extract at 532 nm and 600 nm [44,45].

### 4.4. IAA Measurement

The IAA contents of shoots and roots in tobacco seedlings were measured as described by Sun et al. (2014) and Jia et al. (2018) [16,46]. The samples were grinded with appropriate amount of the antioxidant butyleret hydroxytoluen (BHT) and 80% pre-cooled methanol for 12–16 h. We collected and concentrated the extracted fluid by a rotary evaporator to 10 mL at 40 mL, and then the fluid was extracted with petroleum ether of the same volume. Under a layer liquid it was adjusted to pH 8.5 and added 0.2 g polyvinylpyrrolidone (PVP) then vibrated for 30 min, and then filtered through a 0.45 μm filter over an OASIS HLB (St. Louis, Mo, USA), and chromatographic conditions were described by: Waters 600–2487; Hibar column RT 250 × 4.6 mm; Purospher STARRP-18 (5 μm); column temperature 45 °C; fluid phase: methanol:1% acetic acid (*v/v*, 40/60), isocratic elution; fluid rate: 0.6 mL min^−1^; ultraviolet (UV) detector, *l* = 269 nm; injection volume 20 μL. A 0.22 μm filter was used for filtration of both the buffer and the samples before high-performance liquid chromatography (HPLC) analysis.

### 4.5. Reverse Transcription Polymerase Chain Reaction (qRT-PCR) Analysis

Trizol reagent was used to prepared Total RNAs from the roots and shoots of tobacco seeding. DNase I-treated total RNAs were used for RT by Superscript II. Triplicate quantitative assays were performed with SYBR Premix Ex Taq™ II (Perfect Real Time) kit (TaKaRa Biotechnology, Dalian, China) on the Step One Plus RealTime PCR Systems (Applied Biosystems, Bio-Rad, Berkeley, CA USA). The gene-specific primers for *YUCCAs* and *PINs* family genes of tobacco were used to perform reverse transcription polymerase chain reaction (qRT-PCR) analysis. The primers were shown in Supplemental Table S1. The analysis of relative expression levels used *NtL25* (L18908.1) as internal reference gene and presented as 2^−ΔΔ*C*t^.

### 4.6. Determination of Total P in Plant

Dry samples of about 0.05 g were digested with 5 mL of 98% H_2_SO_4_ and 3 mL of 30% hydrogen peroxide. Then, total P content was analyzed by the molybdate blue method [47].

### 4.7. Measurement of Se and Cd Contents

The comminuted tobacco samples were digested with concentrated HNO_3_ and HClO_4_ (*v/v*, 4:1) [48]. Se and Cd contents were determined by inductively coupled plasma mass spectrometry (ICP-MS 7500A, Agilent, Palo Alto, CA, USA). The accuracy of elemental analysis was verified using standard reference materials from the China Standard Reference Center.

### 4.8. Statistical Analysis

Two-way analysis of variance (ANOVA) and Tukey’s multi-comparisons test (*p* ≤ 0.05) were applied to all data. The results were expressed as the means and the corresponding standard errors. All statistical analyses were completed using the Origin2018 (Origin Lab, Northampton, MA, USA) software.

## 5. Conclusions

This study showed that proper Se supply effectively alleviates the toxicity of Cd in tobacco. Selenium affected the growth of tobacco in the Se–Cd interaction by regulating the expression of the auxin-related genes and enhancing the tolerance to Cd stress by increasing the content of auxin in tobacco. Overexpression of a high-affinity phosphate transporter *NtPT2* increased the content of P and Se and decreased the accumulation of Cd. This study reveals the interaction mechanism of P and auxin in plant growth under the action of Se and Cd and provides new ideas for the safe cultivation of crops in Cd-contaminated soil.

## Supplementary Materials

Supplementary materials can be found at https://www.mdpi.com/1422-0067/20/15/3772/s1.
