# Supplementary material for: Selenium Modulates the Level of Auxin to Alleviate the Toxicity of Cadmium in Tobacco
Source: Int J Mol Sci. 2019 Aug 1;20(15):3772. doi: 10.3390/ijms20153772 (PMC6696094; doi:10.3390/ijms20153772)
Supplement: Supplementary file 1 [file ijms-20-03772-s001.pdf]

Table S1. Primers used to amplify the full-length of *NtPT2* and relative genes for Real-Time PCR.

Gene	**No. ID**	**Primer sequences F (5′ to 3′)**	**Primer sequences R (5′ to 3′)**
NtPT2-Oe	AB042951	CGAGCTCATGTCTGCAGATAACAATC	GCTCTAGATCATTCTTCAGTTATAG
NtPT2	AB042951	GGCGCCTTCATTGCTGCTGTCT	CGCGAACCTTCCAACGATATC
NtPIN1a	KC347302.1	GGAGCTGCAGCACAACAAAGT	ACCTTTCTTGTTATTAGTGC
NtPIN1c	XM_016658576	CTGCTGTTGTGCCACTTTATG	GAAAGAGAGAAGTGGAACTGCG
NtPIN4	KC433529.1	GCAGTCCCTTTACTTTCC	CCATTCTAGGCTACCATTT
NtYUCC6	XM_019373868	GGGTCCAGTAATTGTAGGAGC	TTTGAGTTGCCATAAAGAAGC
NtYUCC8	XM_016592388	ATGTGTATGGGTAAATGGTCC	CAGATTTTTCCAAGATTACAC
NtYUCC9	XM_016635159	GCGAAGATGTGTTTGGGTAA	GCCATAAAGATGCAATACAATC
NtL25	L18908	CCGTCCAAAAAATCTGACCC	TCTTCAAAGTCTTAGGTCGG

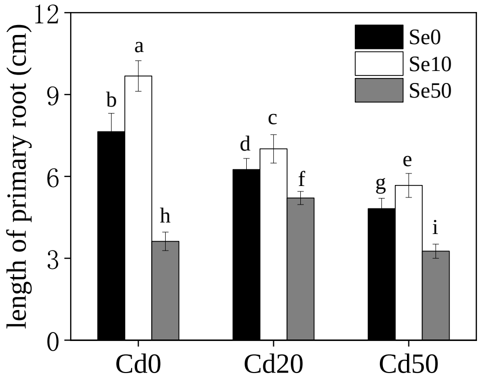

Figure S1. The length of primary root of tobacco under different Se and Cd concentration supply conditions. 14-days-old seedlings were grown in pot with sand under different Se and Cd treatments for 21 days. **Se0**: no Se; **Se10**: Se, 10 μM; **Se50**: Se, 50 μM; **Cd0**: no Cd; **Cd20**: Cd, 20 μM; **Cd50**: Cd, 50 μM. Values are presented as the means ± SD of five biological repeats. Different letters indicate significant differences ($p < 0.05$).

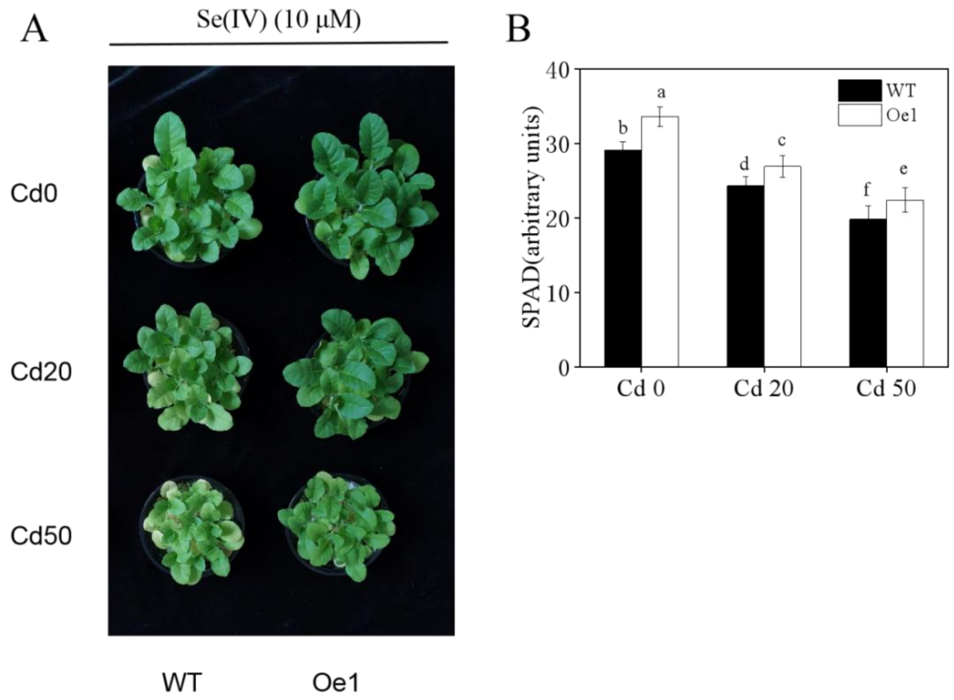

Figure S2. The phenotype and SPAD of tobacco seedlings in Se (10 μM) and different Cd (0, 20, 50 μM) concentrations supply conditions. WT: wild-type tobacco; Oe1: *NtPT2-Oe* transgenic tobacco. 14-days-old seedlings were grown in pot with sand under different Se and Cd treatments for 21 days. **Se0**: no Se; **Se10**: Se, 10 μM; **Se50**: Se, 50 μM; **Cd0**: no Cd; **Cd20**: Cd, 20 μM; **Cd50**: Cd, 50 μM. Values are presented as the means ± SD of five biological repeats. Different letters indicate significant differences ($p < 0.05$).Values are presented as the means ± SD of five biological repeats. Different letters indicate significant differences ($p < 0.05$).

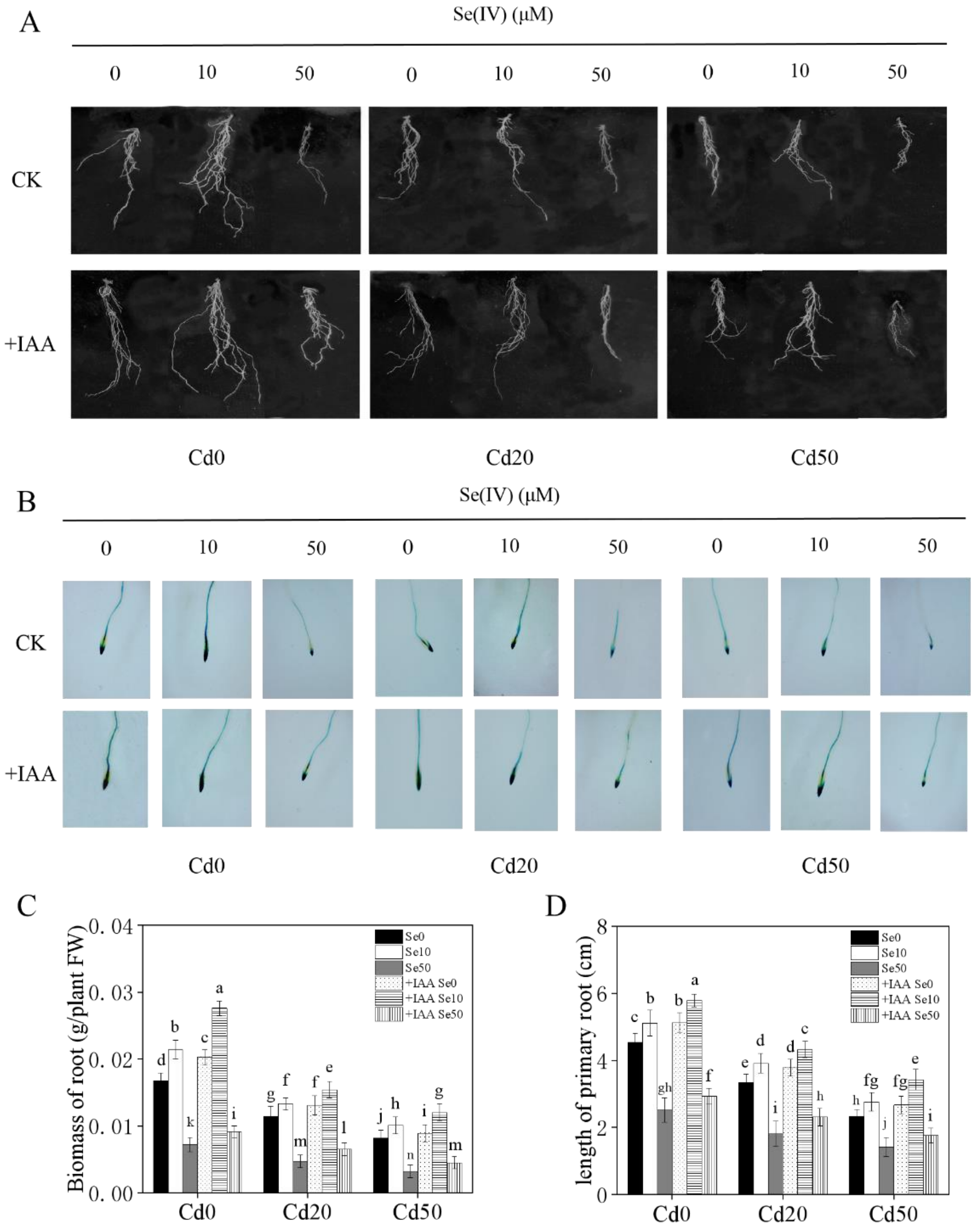

Figure S3. Characterization of root and histochemical localization of *DR5::GUS* transgenic tobacco under Se and Cd supply conditions with adding IAA (100 nM). A, The phenotype of root under different Se and Cd concentration supply conditions with adding IAA (100 nM); B, Histochemical localization of *DR5::GUS* transgenic tobacco under different Se and Cd concentration supply conditions with adding IAA; C, Biomass of root in tobacco under different Se and Cd concentration supply conditions with adding IAA (100 nM), D, Length of primary root of tobacco under different Se and Cd concentration supply conditions with adding IAA (100 nM). 14-days-old seedlings were grown in pot with sand under different Se (0, 10, 50 μM) and Cd (0, 20, 50 μM) concentrations with adding IAA (100 nM) for 7 days. **Se0**: no Se; **Se10**: Se, 10 μM; **Se50**: Se, 50 μM; **Cd0**: no Cd; **Cd20**: Cd, 20 μM; **Cd50**: Cd, 50 μM. **CK:** control, no IAA. Values are presented as the means ± SD of five biological repeats. Different letters indicate significant differences ($p < 0.05$).

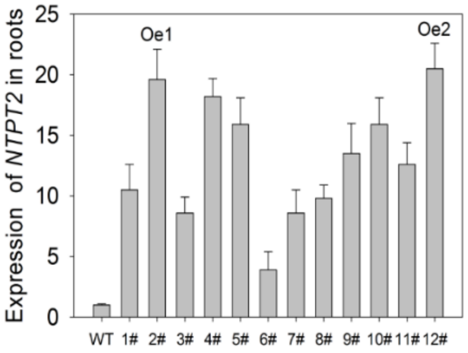

Figure S4. Expression of *NtPT2* in roots of twelve transgenic lines. Values are presented as the means ± SD of five biological repeats. Different letters indicate significant differences ($p < 0.05$).
